# *Drosophila* Rhodopsin 7 can partially replace the structural role of Rhodopsin 1, but not its physiological function

**DOI:** 10.1007/s00359-017-1182-8

**Published:** 2017-05-12

**Authors:** Rudi Grebler, Christa Kistenpfennig, Dirk Rieger, Joachim Bentrop, Stephan Schneuwly, Pingkalai R. Senthilan, Charlotte Helfrich-Förster

**Affiliations:** 1 Neurobiology and Genetics, Biocenter, Theodor Boveri Institute, University of Würzburg, 97074 Würzburg, Germany; 2 Cell- and Neurobiology, Zoological Institute, Karlsruhe Institute of Technology (KIT), Karlsruhe, Germany; 3 Developmental Biology, Institute of Zoology, University of Regensburg, Regensburg, Germany; 4 Oxitec Ltd, 71 Innovation Drive, Milton Park, Oxford OX14 4RQ UK

**Keywords:** Phototransduction, Electroretinograms, Rhodopsins, Compound eyes, *Drosophila melanogaster*

## Abstract

*Rhodopsin 7* (*Rh7*), a new invertebrate *Rhodopsin* gene, was discovered in the genome of *Drosophila melanogaster* in 2000 and thought to encode for a functional Rhodopsin protein. Indeed, Rh7 exhibits most hallmarks of the known Rhodopsins, except for the G-protein-activating QAKK motif in the third cytoplasmic loop that is absent in Rh7. Here, we show that Rh7 can partially substitute Rh1 in the outer receptor cells (R1–6) for rhabdomere maintenance, but that it cannot activate the phototransduction cascade in these cells. This speaks against a role of Rh7 as photopigment in R1–6, but does not exclude that it works in the inner photoreceptor cells.

**Electronic supplementary material:**

The online version of this article (doi:10.1007/s00359-017-1182-8) contains supplementary material, which is available to authorized users.

## Introduction

Vision begins with the absorption of photons by visual pigment molecules. Rhodopsins are membrane-bound G-protein-coupled receptors that absorb photons, undergo conformational changes, and activate a G-protein to initiate visual signal transduction. Animal genomes typically contain multiple *Rhodopsin* genes coding for Rhodopsins with different spectral properties providing the basis for color vision. Each photoreceptor cell usually expresses a single rhodopsin, but exceptions are known in both vertebrates and invertebrates (Applebury et al. 2000; Hu et al. 2011, 2014; Mazzoni et al. 2004; Stavenga and Arikawa 2008). The fruit fly *Drosophila melanogaster* possesses six different well-characterized Rhodopsin molecules, Rh1 to Rh6. With the exception of Rh2, all Rhodopsins are found in the receptor cells of the compound eyes: Rh1 is expressed in the six outer receptor cells (R1–6) of each eye unit and Rh3 to Rh6 are expressed in the two inner receptor cells (R7, R8) (reviewed in Rister et al. 2013; Behnia and Desplan 2015). A seventh Rhodopsin, Rh7, of still unknown location and function was predicted from the genome in 2000 (Adams et al. 2000; Terakita 2005). qPCR studies showed that Rh7 is expressed at low levels in the compound eyes, suggesting that it may be co-expressed with one or several of the other Rhodopsins (Posnien et al. 2012; Senthilan and Helfrich-Förster 2016).

The aim of the present study was to investigate a potential function of Rh7 in the compound eyes. A suited method to reveal the properties of an unknown Rhodopsin is to express it in R1–6 instead of Rh1 (Feiler et al. 1988; 1992; Townson et al. 1998; Salcedo et al. 1999; Knox et al. 2003; Hu et al. 2014). Rh1 is required for proper rhabdomere morphogenesis and maintenance, in addition to its role as photopigment (O’Tousa et al. 1985; Kumar and Ready 1995; Kumar et al. 1997; Zuker et al. 1985). Thus, loss of Rh1 (in *ninaE* ^*17*^ mutants) leads to the collapse of rhabdomeric microvilli inside the photoreceptor cytoplasm (Ahmad et al. 2007; Bentrop 1998; Kurada and O’Tousa 1995; Leonard et al. 1992). This can be prevented by expressing other functional Rhodopsins in R1–6 of *ninaE* ^*17*^ mutants (Kumar et al. 1997). Here, we expressed *Rh7* instead of *Rh1* under the *Rh1* promotor (*Rh1*–*Rh7;ninaE* ^*17*^ flies) and investigated whether Rh7 can (1) rescue the retinal degradation provoked by the *ninaE* ^*17*^ mutation and (2) lead to normal electroretinogram (ERG) responses. We also expressed Rh7 in addition to Rh1 (*Rh1*–*Rh7* flies) to see whether this increases the ERG responses.

## Materials and methods

### Fly strains

Wild-type CantonS (WT_CS_) as well as flies with yellow body color and white eyes (yellow^−^ *white* ^*1118*^ = *y* ^−^ *w* ^*1118*^) served as control for semi-thin sections and immunocytochemistry. For qPCR, deep pseudopupil, and ERG measurements, only flies in the *y* ^−^ *w* ^*1118*^ background were used. In addition, *ninaE* ^*17*^ and *sev* ^*LY3*^ mutants were in the *y* ^−^ *w* ^*1118*^ background. *ninaE (*=*neither inactivation nor afterpotential E)* codes for Rh1 and *y* ^−^ *w* ^*1118*^ *; ninaE* ^*17*^ mutants are *Rh1* null mutants (O’Tousa et al. 1985). *sev (sevenless*) codes for a tyrosin kinase that is critical for the development of the photoreceptor cell R7 (Basler and Hafen 1988) and *y* ^−^ *w* ^*1118*^ *sev* ^*LY3*^ mutants lack the inner photoreceptor cell R7 (Harris et al. 1976). In the following, we will omit “*y* ^−^ *w* ^*1118*^” and simply use *ninaE* ^*17*^ and *sev* ^*LY3*^.

### Generation of *Rh1*–*Rh7* transgenic flies

To generate flies expressing the *Rh7* coding region under control of the *Rh1* promotor, the full-length *Rh7* CDS was amplified by PCR from a commercially available cDNA clone, GH14208 (Berkley *Drosophila* Genome Project), using a primer pair creating restriction enzyme sites for EcoRI or NotI, respectively. The digested PCR product was first ligated into vector pBRh1UTR providing an *Rh1* minimal promoter and the Rh1–3′UTR (kind gift of A. Huber, Universität Hohenheim). From this vector, a NotI/XhoI-fragment containing *Rh1* promotor + *Rh7* coding sequence + *Rh1 3*′-UTR was excised and ligated into the P-element vector Yellow-C4 (P. Geyer, University of Iowa). To create transgenic fly lines, the construct was then microinjected into *y* ^−^ *w* ^*1118*^ embryos resulting in several insertion lines. The insertion lines were tested for *Rh7* expression by qPCR. One line with the transgene insertion on the second chromosome (*y* ^−^ *w* ^*1118*^ *;Rh1*–*Rh7;*+*)* yielded the highest Rh7 expression and was subsequently used for all experiments. This line was crossed into the *ninaE* ^*17*^ mutant background (*y* ^−^ *w* ^*1118*^ *;Rh1*–*Rh7;ninaE* ^*17*^ *)* to get flies that express Rh7 instead of Rh1. Furthermore, the *y* ^−^ *w* ^*1118*^ *;Rh1*–*Rh7;*+ line was backcrossed into the *y* ^−^ *w* ^*1118*^ background for several generations (=backcross) to get control flies with the same genetic background as the transgenics. In the following, we will simply call the lines *Rh1*–*Rh7, Rh1*–*Rh7;ninaE* ^*17*^, and backcross. We checked the presence of all Rhodopsins by qPCR and did not find any differences to wild-type flies.

### PCR

DNA of single flies was extracted in Squishing Buffer (10 mM Tris–HCl pH8.2, 1 mM EDTA, 25 mM NaCl with 2 mg Proteinase K per ml) by incubating for 30 min at 56 °C. After inactivation of the Proteinase K by incubation at 93 °C for 3 min, the genomic DNA was immediately used for the PCR reaction. The latter was carried out in a peqSTAR 96 Universal Thermocycler (peqLab) using the JumpStart REDTaq Ready Mix (Sigma Aldrich) with the following primers (sequences 5′-3′) of the *Rh1* and *cry* genes (the *cry* gene was used as a reference gene):

- *Rh1*: TCTGTATTTCGAGACCTGGGTGCTC; GACATGAACCAGATGTAGGCAATCTTGC
- *cry*: CGGAGTTGATGAATGTCC; GCATGTTTCGCTTTACGG.

### qPCR

The relative mRNA levels of *Rh1* and *Rh7* in different fly strains were quantified via qPCR in 1- and 10-day-old flies as described in Senthilan and Helfrich-Förster (2016). Total RNA was extracted from the retinas of five flies per strain and age using the Quick-RNA™ MicroPrep Kit from Zymo Research and reversely transcribed using the Qiagen QuantiTect Reverse Transcription Kit. qPCRs were then carried out with the Bioline SensiFAST SYBR No-ROX Kit in combination with the Qiagen Rotor-Gene Q machine and 0.1 µM PCR primers. For each strain and tissue, three biological replicates were examined, and for each replicate, two PCRs were run. The relative mRNA levels were calculated using the ΔCT equation and alpha-tubulin was used as the reference gene. To exclude gDNA contamination, we designed our Rhodopsin primers in an intron-spanning way, so that the PCR products obtained from the cDNA and the gDNA will differ by size and by melting temperature.

The following genes and primers (sequences 5′-3′) were used:

- *Rh1*: GGAGTAGAAGATCAGGTATGAGCGTG; TGCCTACATCTGGTTCATGTCGAGC
- *Rh7*: CATCTGCGACTTTCTGATGCTCATC; GGATGCACCACCACATTGTACCGATC
- *Alpha*-*tub*: TCTGCGATTCGATGGTGCCCTTAAC; GGATCGCACTTGACCATCTGGTTGGC.

### Rh7 antibody generation

Two different peptide antibodies against Rh7 were generated: one against a 21-mer peptide in the intracellular domain of Rh7 (aa411-431: TRSSYMTRSRSSFTHRLRTST) and one against an 18-mer peptide in the extracellular domain of Rh7 (aa54-71: TESSAVNVGKDHDKHVND). For the first antibody, the cDNA fragment encoding the intracellular peptide was cloned into the bacterial expression vector pQE40 (Qiagen). The recombinant protein (intracellular peptide coupled to dihydrofolate reductase) was expressed in *Escherichia coli* and used to immunize rabbits. These experiments were registered and conducted according to the legal animal care regulations (RP Karlsruhe, AZ. 35-9185.82/977/99). The second antibody was also generated in rabbits, but by Pineda Antikörper-Service (Berlin, Germany). It was affinity purified using the original peptide bound to Sepharose 6B columns prior to use. Before immunization, serum samples were taken to obtain preimmune serum as negative control for unspecific immunoreactivity. Dot blot analysis was used to confirm the selective binding of Rh7 antibody to the purified peptide.

### Histology and immunocytochemistry

For evaluating the fine-structure of the retina, fly heads were embedded in Epon and 0.2 µm-thick semi-thin sections of the eyes (from distal to proximal) and were cut with a microtome (Leica Ultracut). After mounting on glass slides, the sections were stained with methylene-blue (Romeis 2015). Ten flies of WT controls, *ninaE* ^*17*^ mutants, and *ninaE* ^*17*^ mutants with *Rh7* expression in R1–6 (*Rh1*–*Rh7;ninaE* ^*17*^), respectively, were stained and analyzed at the age of 1, 6, 10 days, and 5 weeks. The rhabdomere size of R1–6 was determined in 10-day-old flies in ImageJ as stated below.

For anti-Rh7, anti-Rh1, and anti-RDHB (visualizing the pigment cells surrounding the photoreceptor cells; Wang et al. 2012) immunocytochemistry, retinas of 4–6-day-old flies were dissected and fixed as described in Hsiao et al. (2012). Primary antibodies were applied for 2 days at room temperature (rabbit anti-Rh7 at 1:100; mouse anti-Rh1 from Developmental Studies Hybridoma Bank at 1:100 and rabbit anti-RDHB, kindly donated by Craig Montell, at 1:100). Fluorescent secondary antibodies (Alexa Fluor 488, 555, 635, Invitrogen) were applied overnight (at 1:1000). Immunostainings were visualized by confocal microscopy (Leica TCS SPE).

### Evaluation of rhabdomere size

Rhabdomere size of 1-day-old flies was determined by measuring the areas of single rhabdomeres in five ommatidia of three different retinas with ImageJ (function “measure” under “analyze”). This was done for R1–6 and R8 of *ninaE* ^*17*^ mutants, *Rh1*–*Rh7;ninaE* ^*17*^ and *Rh1*–*Rh7* flies, respectively. In all cases, cross sections at the level of R8 were taken for the measurements. The sizes of R1–6 were averaged for each ommatidium. A mean size for a typical R1–6 rhabdomere was then determined by averaging the values for all 15 ommatidia.

### Deep pseudopupil

The deep pseudopupil was determined in vivo in 6-day-old white-eyed flies under white orthodromic illumination as described in Franceschini and Kirschfeld (1971). Flies were anaesthetized and glued to a glass slide with the left eye oriented to the top and placed under a stereo microscope. The eye was illuminated with a cold light source (KL 1500 LED plus, Leica). The objective (Planapo 1.6×, n:A. 0.24) was focused onto a region below the crystal cones at which R7 was seen best.

### ERG recordings

**Fig. 1:**
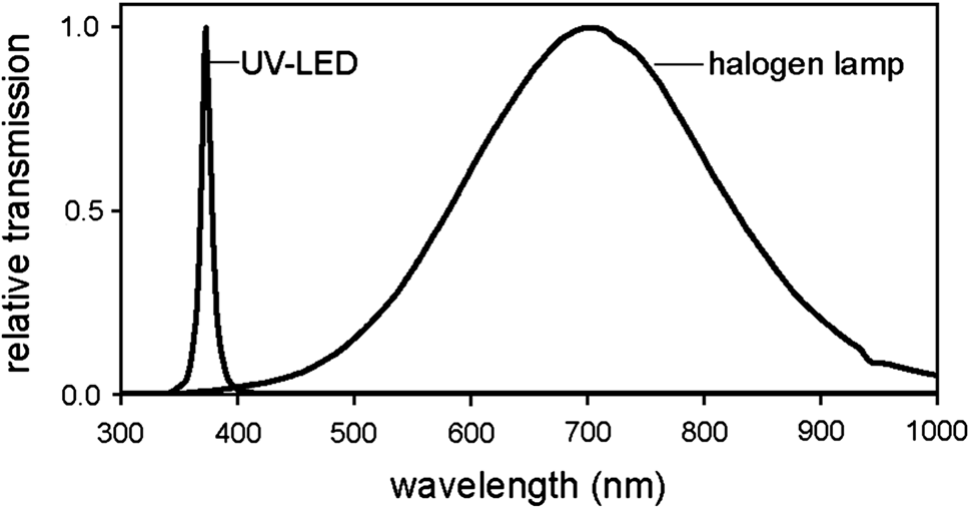
Relative emission spectra of the light sources used to provoke electroretinograms (ERGs). Maximal emission is normalized to 1

ERG recordings were performed in 6- and 10-day-old flies at 20 °C as stated in Mazzotta et al. (2013). Before each experiment, flies were dark-adapted for 15 min. A halogen lamp (Spindler & Hoyer) was used for the generation of white light stimuli of 400 ms duration and different intensities. The halogen lamp emits light between 380 and 1000 nm as measured by a QE6500 spectrophotometer (Fig. 1). For generating light in the UV range, an LED with a *λ*_max_ of 370 nm (Roithner) was used (Fig. 1). To keep the flies in a reasonably dark-adapted state, the inter-stimulus interval was 20 s. Experiments were run starting with the lowest light intensity to minimize adaptation effects. Maximum light intensity was 9.75 × 10^14^ photons cm^−2^ s^−1^. The receptor potential amplitudes of the electroretinogram (ERG) responses were plotted as a function of the related light intensity to yield irradiance response curves. Each curve was obtained from *n* = 7–13 flies. The sum of the measured receptor potential amplitudes for all light intensities were calculated for each single fly and statistically compared between different strains.

## Results

### Low expression levels of Rh7 in the retina can be enhanced by ectopic expression in R1–6

**Fig. 2:**
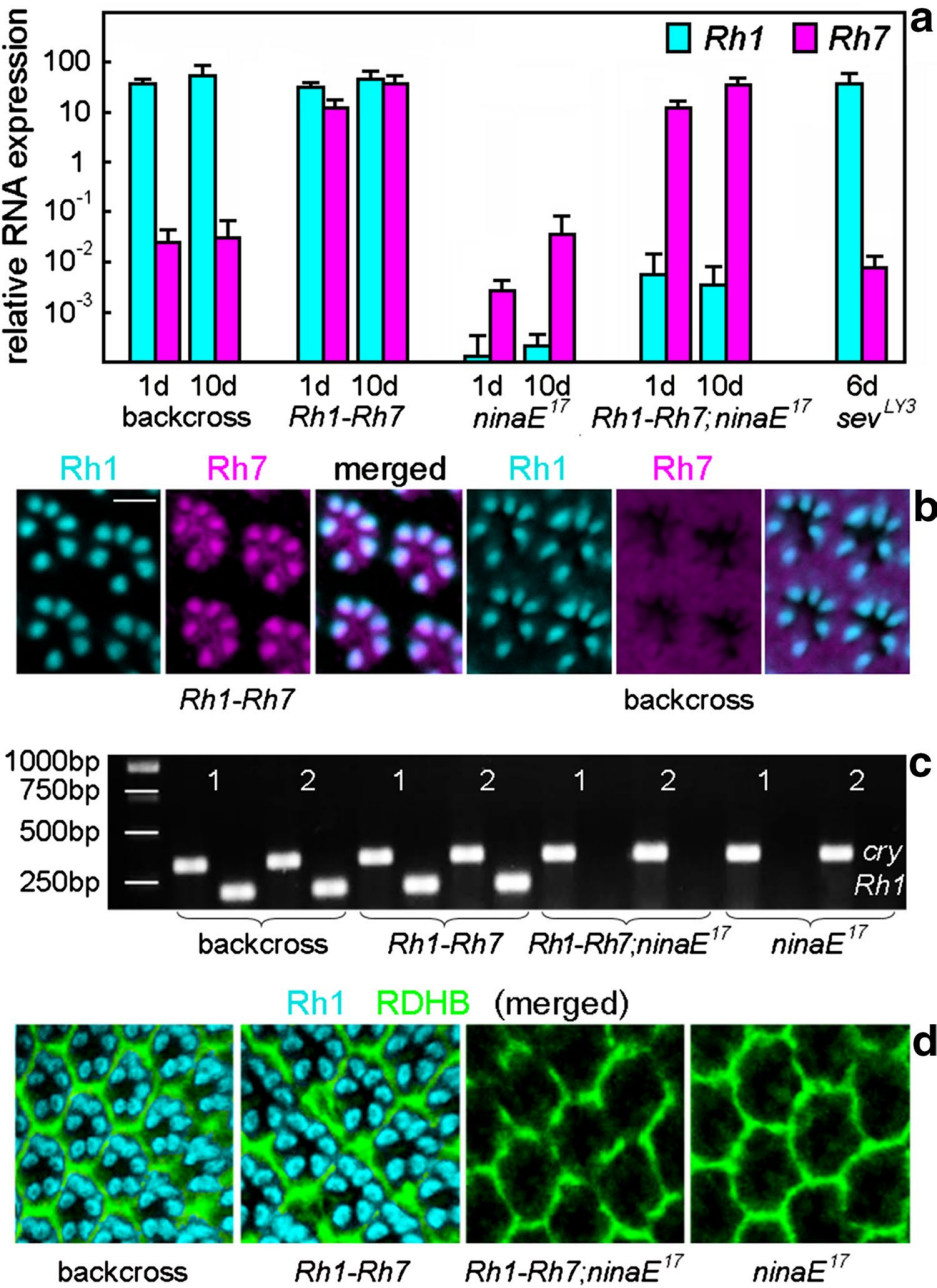
*Rh7* and *Rh1* expressions in the compound eyes. **a** qPCRs of *Rh1* and *Rh7* expressions (*cyan* or *magenta bars*, respectively) in the retina of 1- and 10-day-old flies, and in case of *sev* ^*LY3*^ of 6-day-old flies. All flies are in a white-eyed *y* ^−^ *w* ^*1118*^ background. For details, see text. **b** Anti-Rh1 and anti-Rh7 double labeling in whole-mount retinas of *Rh1*–*Rh7* and control flies (*backcross*). Rh1 is visualized in cyan and Rh7 in *magenta*. Whereas no specific Rh7 staining is visible in the R1–6 rhabdomeres of control flies, the Rh7 antibody labels all R1–6 rhabdomeres of *Rh1*–*Rh7* flies. These stainings are obtained with the anti-Rh7 antibody raised against the 18-mer peptide in the extracellular domain (aa54-71) of Rh7. *Scale bar* 5 µm. **c** PCR products of *Rh1* and *cryptochrome* (*cry*) genes from two flies (1, 2) per genotype, respectively. *ninaE* ^*17*^ mutants lack the PCR product of *Rh1*. **d** Anti-Rh1 and anti-RDHB (retinol dehydrogenase B) double labeling in whole-mount retinas of the different genotypes. RDHB marks the pigments cells that surround the photoreceptor cells of each ommatidium. *ninaE* ^*17*^ mutants lack Rh1 labeling

In comparison with *Rh1*, *Rh7* was expressed at rather low levels in the retina of the control flies (backcross) (<1:1000, Fig. 2a). As soon as under control of the *Rh1* promotor (*Rh1*–*Rh7* or *Rh1*–*Rh7;ninaE* ^*17*^ flies), *Rh7* levels rose to the level of retinal *Rh1* expression (Fig. 2a). Thus, ectopic Rh7 expression led to high levels of *Rh7* mRNA. Immunostaining with our Rh7 antibodies showed that the *Rh7* mRNA was also translated into Rh7 protein. Whereas the Rh7 antibodies could not detect Rh7 in the retina of wild-type flies, Rh7 was clearly detectable in R1–6 of *Rh1*–*Rh7* flies (Fig. 2b). Both Rh7 antibodies yielded the same results. Staining in wild-type flies (WT_CS_, y^−^w^1118^ and the backcross) was quite variable and sometimes gave a uniform background staining of all tissues (see Fig. 2b) and sometimes staining in the interrhabdomeric space, as can be seen in the retina of the *Rh1*–*Rh7* flies, as shown in Fig. 2b. However, the rhabdomeres of the photoreceptor cells were never labeled in wild-type flies, suggesting that Rh7 levels of wild-type flies are below the detection limit. This coincides with the low mRNA expression levels of *Rh7* (Fig. 2a).

*ninaE* ^*17*^ mutants carry a 1.6 kb deletion in the 5′ region of the *Rh1* gene with no detectable *Rh1* transcript on an agarose gel (O’Tousa et al. 1985). As expected, the genomic DNA PCR yielded no visible *Rh1* gene product on the gel (Fig. 2c), while using qPCR, which is more sensitive, we still detected marginal mRNA signals in the *ninaE* ^*17*^ mutants (Fig. 2a). This residual expression is probably a result of the incomplete deletion in the *Rh1* gene. Residual *Rh1* expression was elevated after expressing *Rh7* in R1–6 of *ninaE* ^*17*^ mutants (Fig. 2a) most probably because *Rh7* expression prevented the degeneration of R1–6 (see below). Nevertheless, the Rh1 protein was not detectable at all in *ninaE* ^*17*^ mutants nor in *Rh1*–*Rh7;ninaE* ^*17*^ mutants (Fig. 2d). This coincides with previous studies showing that *ninaE* ^*17*^ mutants lack a functional Rh1 protein and they are, therefore, considered as *Rh1* null mutants (Bentrop et al. 1997).

Most interestingly, the degeneration of R1–6 in 10-day-old *ninaE* ^*17*^ mutants did not reduce *Rh7* mRNA levels (Fig. 2a), suggesting that Rh7 is normally not expressed in R1–6. Furthermore, *Rh7* is still present in *sevenless* ^*LY3*^ (*sev* ^*LY3*^) mutants that lack photoreceptor cell R7 (Fig. 2a) indicating that *Rh7* is also not expressed in R7 and pinpointing to an expression of *Rh7* in R8. Indeed, a reporter line carrying the gene for the green fluorescent protein (GFP) in the first intron of the *Rh7* gene showed weak GFP expression in R8 (Kistenpfennig et al. 2017, in preparation).

### Rh7 can partially replace Rh1 in maintaining rhabdomere structure of R1–6

**Fig. 3:**
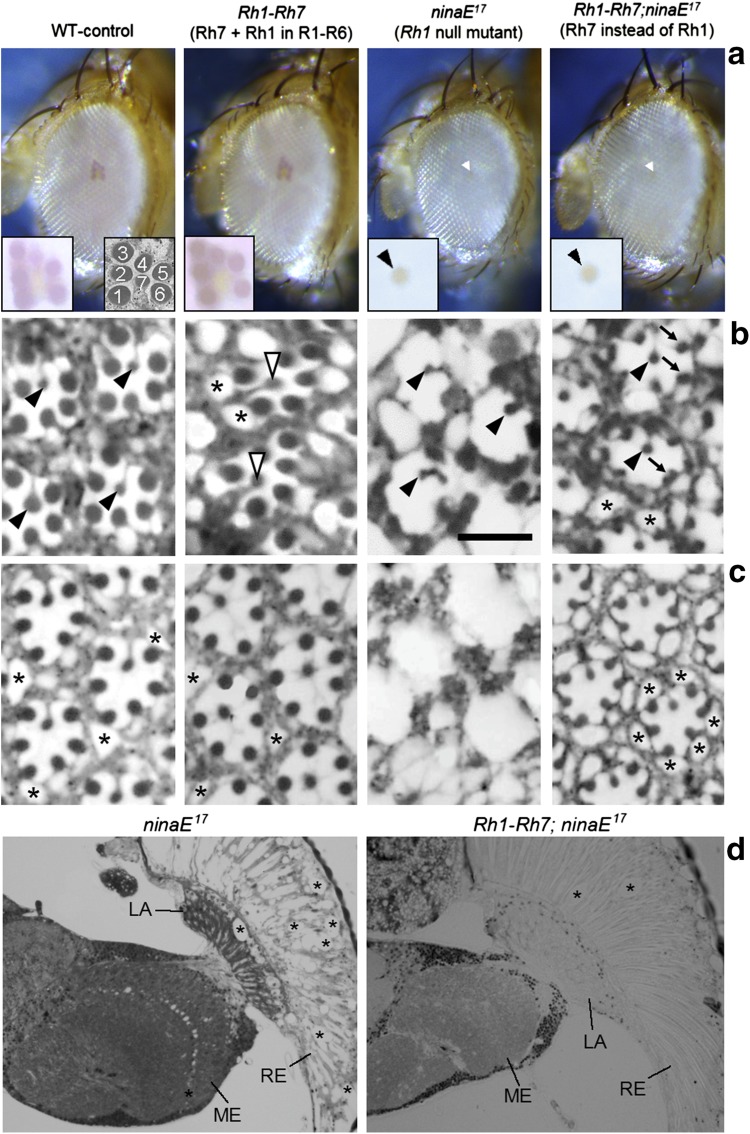
Rh7 can partially rescue maintenance of Rh1 in of R1–6. **a** Deep pseudopupil in the eyes of 6-day-old WT-control flies (*y* ^−^ *w* ^*1118*^), flies that express *Rh7* in addition to *Rh1* in the outer receptor cells (*Rh1*–*Rh7*), *Rh1* null mutants (*ninaE* ^*17*^), and flies that express *Rh7* instead of *Rh1* (*Rh1*–*Rh7;ninaE* ^*17*^). An enlarged image of the deep pseudopupil is shown in the insets at the *left bottom* of each picture. The arrangement and numbering of the rhabdomeres are shown in the inset at the right bottom of the WT-control picture. Only control flies and *Rh1*–*Rh7* flies show a regular deep pseudopupil indicating a correct arrangement of R1–6 and their rhabdomeres. In *ninaE* ^*17*^ and *Rh1*–*Rh7;ninaE* ^*17*^ flies, only the inner receptor cells R7 and R8 show a weak yellowish pseudopupil (*arrowhead*). All flies are in a white-eyed background (applies also for **b**–**d**). **b** Semi-thin sections of retinas of 10-day-old flies (the same fly strains, as depicted in Fig. 2a). *Arrowheads* point to photoreceptor cell 8 that are present in all ommatidia of WT control, *Rh1*–*Rh7* and *Rh1*–*Rh7;ninaE* ^*17*^ flies (together with photoreceptor cell 7 highlighted by open arrowheads). In *Rh1* null mutants (*ninaE* ^*17*^), receptor cells R7/8 are present in ~60% of the ommatidia. Receptor cells R1–6 degenerate in *ninaE* ^*17*^ mutants and are completely absent in all ommatidia. Flies that express *Rh7* instead of *Rh1* still retain small rhabdomeres of R1–6 (*arrows*). Flies that express *Rh7* in addition to *Rh1* have normally sized rhabdomeres, but they show holes (exemplary marked by *asterisks*) between the rhabdomeres, suggesting that an excess of rhodopsin disturbs the general morphology of the retina. *Scale bar* 5 µm. **c** Semi-thin sections of retinas of 5-week-old flies. *ninaE* ^*17*^ mutants lack all photoreceptors and rhabdomeres, whereas these were still present in *Rh1*–*Rh7;ninaE* ^*17*^ flies. The retina of all flies, including the WT controls, show some degeneration visible by the holes between the ommatidia (*asterisks*). **d** Horizontal semi-thin sections of the right retina, lamina and medulla of 5-week-old *ninaE* ^*17*^ and *Rh1*–*Rh7;ninaE* ^*17*^ flies, respectively. In *ninaE* ^*17*^ mutants the retina (RE) is filled with holes (*asterisks*) and lost its usual regular structure, the lamina (LA) is rather thin and also full of holes and the medulla (ME) contains holes in its distal part, where R7 and R8 end. In contrast, *Rh1*–*Rh7;ninaE* ^*17*^ flies retain the regular structure of the retina [only 2 holes are visible in this section (*asterisks*)], the lamina is thicker than that of *Rh1*–*Rh7;ninaE* ^*17*^ flies and the distal medulla is virtually free of holes. Please note that the stronger staining of the *ninaE* ^*17*^ semi-thin section is also typical for degenerating tissue (Kretschmar et al. 1997)

To test whether Rh7 can rescue the retinal degradation provoked by the *ninaE* ^*17*^ mutation, we first analyzed the deep pseudopupil of 6-day-old flies without Rh1, with Rh7 instead of Rh1, and with Rh1 and Rh7 in R1–6. The deep pseudopupil is an optical phenomenon that comes about by superposition of the virtual images of the rhabdomeres of neighboring ommatidia (Franceschini and Kirschfeld 1971). The deep pseudopupil can only be seen when all rhabdomeres are regularly arranged. Consequently, it disappears in *ninaE* ^*17*^ mutants, in which the rhabdomeres collapse (Fig. 3a). We found the deep pseudopupil in control flies as well as in flies that express *Rh1* and *Rh7*, but not in flies that express *Rh7* instead of *Rh1* (Fig. 3a). This indicates that the rhabdomeres are not regularly arranged in flies that express *Rh7* instead of *Rh1* suggesting that Rh7 cannot rescue retinal degeneration in flies lacking Rh1.

To investigate whether the missing deep pseudopupil is indeed caused by rhabdomere degeneration or just by an irregular arrangement of the rhabdomeres, we additionally performed semi-thin sections of the fly retina. One day after eclosion, the rhabdomeres were still clearly visible in homozygous *ninaE* ^*17*^ mutants, independent of the presence or absence of Rh7 (not shown). However, 6 and 10 days after eclosion, the rhabdomeres of R1–6 were completely absent in *ninaE* ^*17*^ mutants, whereas we still saw small rhabdomeres of R1–6 in the ommatidia of *Rh1*–*Rh7;ninaE* ^*17*^ flies and normal rhabdomeres in the ommatidia of *Rh1*–*Rh7* (Fig. 3b). The R1–6 rhabdomeres of *Rh1*–*Rh7;ninaE* ^*17*^ flies had half the size of wild-type rhabdomeres, whereas the R8 rhabdomeres were of equal size in all strains (Table 1). Besides the size differences of the R1–6 rhabdomeres, we observed holes between the ommatidia in *Rh1*–*Rh7* and *Rh1*–*Rh7;ninaE* ^*17*^ flies that were not visible in controls (asterisks in Fig. 3b). Such holes are indicative of ongoing neurodegeneration (Kretschmar et al. 1997) and have been already described for mutants lacking Rh1 (Kurada and O’Tousa 1995; Bentrop et al. 1997) as well as for flies expressing overactive Rh1 (Iakhine et al. 2004; reviewed by Shieh 2011). Rhabdomeres of R7 and R8 were always visible in the ommatidia of *Rh1*–*Rh7;ninaE* ^*17*^ flies, whereas they were present in 95 and 60% of the ommatidia in 6- and 10-day-old *ninaE* ^*17*^ mutants, respectively. The latter observation indicates that even the inner photoreceptor cells degenerate with increasing age in *ninaE* ^*17*^ mutants. Thus, *Rh7* appears to prevent not only the degeneration of R1–6 but also that of R7/R8.

**Table 1:** Mean area of a rhabdomere determined from semi-thin cross sections of three retinas (5 ommatidia, each) at the level of R8 of 10-day-old flies

Genotype	R1–6 (µm^2^)	R8 (µm^2^)
WT	0.91 ± 0.03	0.47 ± 0.03
*Rh1*–*Rh7*	0.92 ± 0.02	0.47 ± 0.02
*Rh1*–*Rh7;ninaE* ^*17*^	0.47 ± 0.02	0.47 ± 0.02
*ninaE* ^*17*^	–	0.42 ± 0.02

In 5-week-old flies, the differences between *ninaE* ^*17*^ mutants and *Rh1*–*Rh7;ninaE* ^*17*^ flies became most evident. Whereas *ninaE* ^*17*^ mutants of that age showed a severe degeneration of the entire retina and lacked all rhabdomeres, *Rh1*–*Rh7;ninaE* ^*17*^ flies still retained small rhabdomeres in R1–6 and rather normal rhabdomeres of R7 and R8 (Fig. 3c). Holes between the rhabdomeres were now visible even in control flies, but occurred still more frequently in *Rh1*–*Rh7;ninaE* ^*17*^ flies (asterisks in Fig. 3c). In *ninaE* ^*17*^ mutants, even the target neuropils of the photoreceptor cells degenerated. This was most evident in the lamina, in which the axons of R1–6 terminate, and to a lesser degree in the medulla, in which the axons of R7 and R8 terminate (Fig. 3d). In contrast, *Rh1*–*Rh7;ninaE* ^*17*^ flies showed no such signs of degeneration in the lamina and medulla (Fig. 3d).

We conclude that Rh7 can partially replace Rh1 in its rhabdomere and photoreceptor maintenance role. R1–6 photoreceptor cells and their rhabdomeres were present in *Rh1*–*Rh7;ninaE* ^*17*^ flies, but they were significantly smaller than in control flies and obviously not arranged in a sufficient regular manner to enable a deep pseudopupil. This irregular arrangement is hard to see on our semi-thin sections even not at a bigger field of the retinal section (Fig. S1). Nevertheless, one must confess that a precise superposition depends on a formidable regularity in the architecture of the compound eye, such as a constancy of the center-to-center distance between the seven rhabdomere distal endings (Franceschini 1972). Already, small deviations may result in a failure to detect a deep pseudopupil.

### Rh7 cannot rescue the electroretinogram (ERG) of *ninaE*^*17*^ mutants

**Fig. 4:**
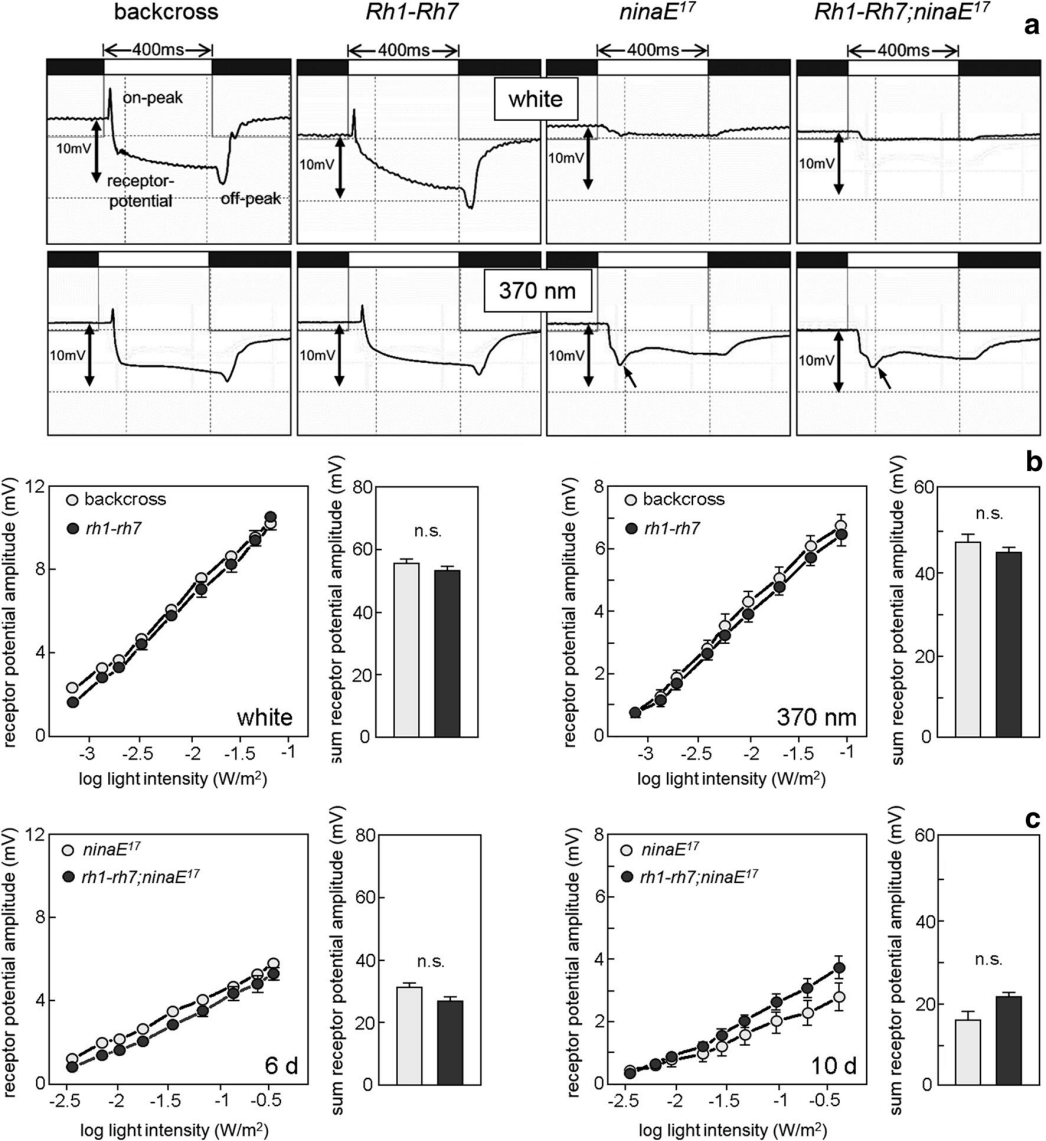
Ectopic Rh7 in R1–6 does not affect the ERG responses. **a** ERGs to white and UV (370 nm) light-pulses of 400 ms of 6-day-old WT-control flies (*backcross*), flies that express *Rh7* in addition to *Rh1* in the outer receptor cells (*Rh1*–*Rh7*), *Rh1* null mutants (*ninaE* ^*17*^) and flies that express *Rh7* instead of *Rh1* (*Rh1*–*Rh7;ninaE* ^*17*^). Control flies and flies expressing *Rh7* in addition to *Rh1* show normal ERG responses with lights-on and lights-off transients and a receptor potential of ~10 mV in between the two. *Rh1* null mutants and flies that express *Rh7* instead of *Rh1* lack the lights-on and lights-off responses that depend on R1–6 and show a significantly lower ERG amplitude. Under UV light, *ninaE* ^*17*^ and *Rh1*–*Rh7;ninaE* ^*17*^ mutants exhibit a small overshoot in their receptor potential (*small arrow*). **b** Amplitude of the receptor potential dependent on log irradiance (dose–response) in backcross and *Rh1*–*Rh7* flies under white and UV light. The *columns to the right* of the dose–response curves indicate the summed receptor amplitudes used for statistical comparison. **c** Amplitude of the receptor potential in 6- and 10-day-old *ninaE* ^*17*^ and *Rh1*–*Rh7;ninaE* ^*17*^ flies. The presence of Rh7 does not cause any differences in the ERG amplitude of 6-day-old flies. In 10-day-old flies, *ninaE* ^*17*^ mutants tend to show lower responses, probably due to the degeneration of photoreceptor cells R7/8 (for details see text)

Since Rh7 can partially rescue rhabdomere degeneration in *ninaE* ^*17*^ mutants, it may also be able to activate the phototransduction cascade upon light stimulation. To test this, we performed extracellular ERG measurements on 6- and 10-day-old white-eyed *ninaE* ^*17*^ and *Rh1*–*Rh7;ninaE* ^*17*^ flies as well as 6-day-old white-eyed control flies (backcross) and flies that express *Rh1* and *Rh7* in R1–6 (Fig. 4). The ERG represents the summed responses of all retinal cells plus the postsynaptic neurons in the lamina to light. There are two primary features of the ERG: the maintained component (or the receptor potential), which results from the activity in the retina, and the on- and off transients, which occur due to postsynaptic activity in the lamina (Pak et al. 1969) (Fig. 4a). R1–6 are the only cells that transmit the evoked signals to the lamina. Therefore, the on- and off transients are absent in *ninaE* ^*17*^ mutants that lack functional R1–6. Furthermore, the receptor potential is rather low in *ninaE* ^*17*^ mutants, since it consists only of the light responses evoked in R7 and R8. We found that the ERG of 6-day-old *Rh1*–*Rh7;ninaE* ^*17*^ flies was not significantly different from *ninaE* ^*17*^ mutants, neither in shape nor in amplitude: The on- and off transients were completely absent (Fig. 4a) and the residual receptor potential in response to increasing light intensity was not significantly different from that of *ninaE* ^*17*^ mutants (Fig. 4c). The same was true under UV light. Only the shape of the ERGs under UV slightly differed from that under white light (Fig. 4a): R1–6 of control and Rh1–Rh7 flies responded to UV, so rapidly that the amplitude of the on transient was lowered. Furthermore, in *ninaE* ^*17*^ and *Rh1*–*Rh7;ninaE* ^*17*^ flies, which lacked on- and off transients, a small overshoot of the receptor potential was visible (arrows in Fig. 4a). These differences were already reported earlier (Bentrop et al. 1997). Nevertheless, even UV light could not provoke higher receptor potentials in flies that expressed R7 instead of Rh1 or in addition to Rh1 (Fig. 4a, b). We conclude that Rh7 is not able to activate the phototransduction cascade in R1–6 and, thus, is not a functional rhodopsin in these cells. The receptor potential present in *ninaE* ^*17*^ and *Rh1*–*Rh7;ninaE* ^*17*^ mutants appears to originate entirely from R7/R8. This is supported by the ERG response curves of 10-day-old *ninaE* ^*17*^ mutants, in which our histological analysis revealed a beginning degeneration of R7/R8: Their ERG response showed a clear tendency to decrease (Fig. 4c).

## Discussion

Many animals express more than one Rhodopsin molecule in their eyes to enhance light sensitivity and to enable color vision. Mosquitoes express even several rhodopsins in one photoreceptor cell (R7) making them responsive to a broad spectrum of visible and UV light and enabling them to be active at very dim light (Hu et al. 2011, 2014). The fruit fly *D. melanogaster* possesses six well-characterized Rhodopsins and a seventh Rhodopsin (Rh7) with unknown function that might be co-expressed with the others. Rh7 seems to be an ancient type of Rhodopsin that is conserved among different arthropods, including species as *Limulus polyphemus* and *Daphnia pulex* (Senthilan and Helfrich-Förster 2016). Rh7 possesses nearly all important features for a visual functional protein, but it lacks the highly conserved QAKK motif, which is, together with the DRY motif, important for G-protein binding and consequently for the activation to the G-protein-coupled cascade. Therefore, it is unknown, whether it is a functional Rhodopsin. Here, we tested whether Rh7 can replace the function of Rh1 by expressing it instead of Rh1 in R1–6.

We found that Rh7 can partially overtake the functions of Rh1. It can fairly replace Rh1 in its rhabdomere maintenance function, but it cannot activate the phototransduction cascade in R1–6, at least not under our experimental conditions. We cannot exclude that Rh7 is a UV-sensitive Rhodopsin with a sensitivity maximum below 320 nm. Our halogen lamp emits almost no light at wavelengths below 400 nm (Fig. 1) and the UV-LED with *λ*_max_ at 370 nm cannot excite Rhodopsins with sensitivity maxima below 320 nm given that the half width of a typical Rhodopsin absorption spectrum is ~100 nm (Salcedo et al. 1999). It is possible that Rh7 is a UV-sensitive Rhodopsin, because it retains primary protein sequence motifs that are characteristic of invertebrate UV pigments, such as the lysine at position 168 (position 110 in Rh1) and the DRY motif (Salcedo et al. 2003; Hu et al. 2014), but it remains questionable that Rh7 can have *λ*_max_ below 320 nm. *Drosophila* UV Rhodopsins, Rh3 and Rh4, have sensitivity maxima at 345 and 375 nm, respectively (Feiler et al. 1992) and other invertebrate UV Rhodopsins are sensitive in the same wavelength range (Popp et al. 1996; Smith et al. 1997; Chase et al. 1997; Salcedo et al. 1999). On the other hand, there are also data supporting the notion that Rh7 is sensitive to visible light, because it contains a tyrosine at position 191 that is also present in all other visible-light-detecting Rhodopsins, such as Rh1, Rh2, Rh5, and Rh6, but changed into a phenylalanine in the UV-sensitive Rh3 and Rh4 (Chou et al. 1996). In sum, it is unlikely that we have been unable to excite Rh7 with our lighting system.

Furthermore, we cannot completely exclude that Rh7 shows extremely weak and slow ERG responses as found for the mosquito Rh7 ortholog Op10 when expressed in R1–6 of *D. melanogaster* (Hu et al. 2014). Hu et al. (2014) used a modified *norpA;ninaE* ^*17*^ mutant background, in which *norpA* is activated only in R1–6. The *norpA* gene codes for the phospholipase C-β, which is an important component in the phototransduction cascade of all *Drosophila* photoreceptor cells (Bloomquist et al. 1988). Eliminating *norpA* in all photoreceptor cells except for R1–6 allowed measuring the activity of the ectopically expressed Op10 without interference from the R7 and R8 cells. In our experiments, R7 and R8 were still active and are responsible for the small receptor potential in *ninaE* ^*17*^ and *Rh1*–*Rh7;ninaE* ^*17*^ mutants (Fig. 4). These responses might have masked putative weak responses of Rh7. Nevertheless, we do not think that this is likely for the following reasons: (1) we compared the ERG responses between *ninaE* ^*17*^ and *Rh1*–*Rh7;ninaE* ^*17*^ mutants at different light intensities. Thus, at high intensities, we should have seen even small differences provoked by Rh7. (2) The activity of R1–6 provokes on- and off transients in the lamina that were clearly visible in the ERGs of WT flies, but completely absent in *ninaE* ^*17*^ mutants (Fig. 4). In none of the measured ERGs of *Rh1*–*Rh7;ninaE* ^*17*^ mutants, on- and off transients were present. This clearly indicates that Rh7 cannot activate the phototransduction cascade in R1–6.

The inability of Rh7 to activate the phototransduction cascade in R1–6 does not mean that Rh7 is not a functional photopigment. It may signal via a different phototransduction cascade that is absent in R1–6. Our data indicate that Rh7 is normally not expressed in R1–6. From sequence alignment, Rh7 is closely related to *Drosophila Rh3, Rh4,* and *Rh5* genes (Senthilan and Helfrich-Förster 2016), suggesting that it is rather expressed in the inner instead the outer receptor cells, and we have first indications that Rh7 is expressed in the inner photoreceptor cell R8 (Kistenpfennig et al. in preparation). In mosquitos, the ortholog of *Drosophila* Rh7, Op10, is expressed in the inner receptor cell R7 together with Op8 (Hu et al. 2014). Although Op10 was able to activate the phototransduction cascade in R1–6 of *D. melanogaster*, it could do so only very weakly, suggesting that it usually operates with different signaling molecules that are not present in R1–6 (Hu et al. 2014). That the inner photoreceptor cells of *D. melanogaster* may use a different phototransduction cascade that works independently from the phospholipase C-ß which has been suggested by previous studies concerning circadian photoreception (Veleri et al. 2007; Szular et al. 2012).

It is also possible that Rh7 signals via a second Rhodopsin present in the inner photoreceptor cells. G-protein-coupled receptors (GPCRs), including Rhodopsin-like GPCRs, can form dimers (Gurevich and Gurevich 2008a, b; Hiller et al. 2013). The assumption that Rh7 may interact with another Rhodopsin to exert its function is supported by the fact that Rh7 and its homologues lack the highly conserved QAKK motif, which is, together with the DRY motif, important for G-protein binding and consequently for the activation of the G-protein-coupled cascade (see above). Thus, Rh7 might signal via a second Rhodopsin and not by itself. Most interestingly, Rh7 has unusually long C- and N-terminal tails that are well suited for the interaction with other proteins (Senthilan and Helfrich-Förster 2016).

At present, we cannot answer the question, in which photoreceptor cell Rh7 is working and what is the way of its action. Its presence in most groups of arthropods, excluding those living in aphotic environments, suggests that it has a conserved function in light signaling either as visual or as non-visual Rhodopsin (Senthilan and Helfrich-Förster 2016). Further studies are needed to answer this interesting question.

## Electronic supplementary material

Below is the link to the electronic supplementary material.

Supplementary material 1 (PDF 128 kb)
