# Supplementary material for: Drosophila Rhodopsin 7 can partially replace the structural role of Rhodopsin 1, but not its physiological function
Source: J Comp Physiol A Neuroethol Sens Neural Behav Physiol. 2017 May 12;203(8):649–59. doi: 10.1007/s00359-017-1182-8 (PMC5537319; doi:10.1007/s00359-017-1182-8)
Supplement: Supplementary file 1 — Supplementary material 1 (PDF 128 kb) [file 359_2017_1182_MOESM1_ESM.pdf]

JOURNAL OF COMPARATIVE PHYSIOLOGY A

Drosophila **Rhodopsin 7 can partially replace the structural role of Rhodopsin 1, but not its physiological function**

Rudi Grebler[1], Christa Kistenpfennig[1], Dirk Rieger[1], Joachim Bentrop[2], Stephan Schneuwly[3], Pingkalai R. Senthilan[1], Charlotte Helfrich-Förster[1*]

[1] Neurobiology and Genetics, Biocenter, University of Würzburg, 97074 Würzburg, Germany

[2] Cell- and Neurobiology, Zoological Institute, Karlsruhe Institute of Technology (KIT), Karlsruhe, Germany

[3] Developmental Biology, Institute of Zoology, University of Regensburg, Regensburg, Germany

[+] present address: Oxitec Ltd, 71 Innovation Drive, Milton Park, Oxford, OX14 4RQ, UK

*E-mail of corresponding author:

charlotte.foerster@biozentrum.uni-wuerzburg.de

Supplementary Figure 1

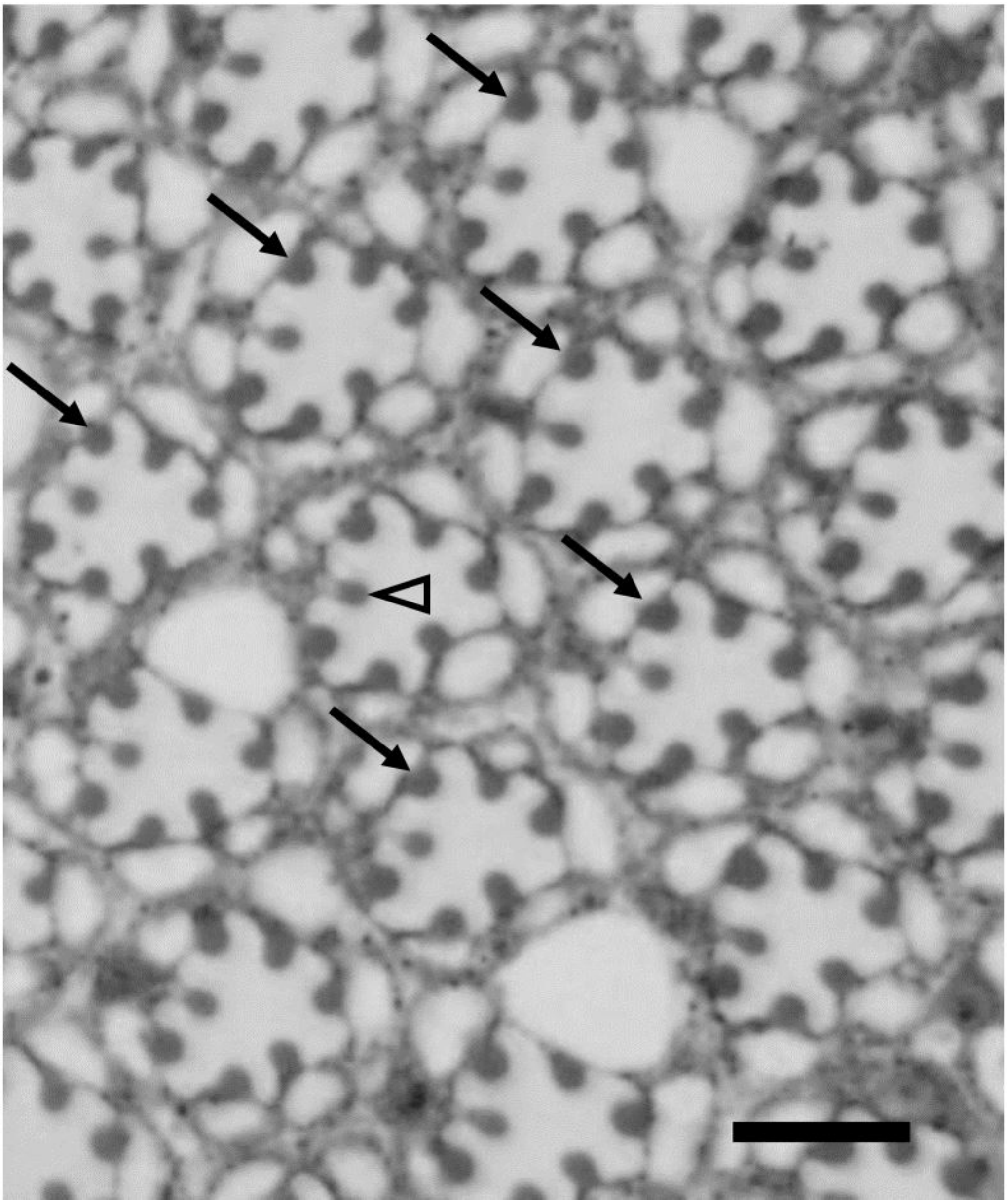

Figure S1. Flies with Rh7 instead of Rh1 in photoreceptors R1-6 maintain a quite regular structure of the rhabdomeres.
Semi-thin sections of a retina of a 10d old *Rh1-Rh7;ninaE^{17}* fly. Arrowheads point to photoreceptor cells 1 in 6 neighboring ommatida; photoreceptor cell 7 is highlighted in the central ommatidium by an open arrowhead. Scale bar: 5 µm.
